# Quantitative study of ^18^F-(+)DTBZ image: comparison of PET template-based and MRI based image analysis

**DOI:** 10.1038/s41598-018-34388-6

**Published:** 2018-10-30

**Authors:** Hsu Jung Lung, Yi-Hsin Weng, Ming-Ching Wen, Ing-Tsung Hsiao, Kun-Ju Lin

**Affiliations:** 1 Department of Neurology, Linkou Medical Center, Chang Gung Memorial Hospital and College of Medicine, Chang Gung University, Taoyuan, Taiwan; 2 Taipei Medical University, Graduate Institute of Humanities in Medicine and Research Center for Brain and Consciousness, Shuang Ho Hospital, Taipei, Taiwan; 3 Department of Neurology, National Neuroscience Institute, Singapore, Singapore; 4 Department of Nuclear Medicine and Center for Advanced Molecular Imaging and Translation, Linkou Chang Gung Memorial Hospital, Taoyuan, Taiwan; 5 Department of Medical Imaging and Radiological Sciences and Healthy Aging Research Center, Chang Gung University, Taoyuan, Taiwan

## Abstract

[^18^F]9-fluoropropyl-(+)-dihydrotetrabenazine (^18^F-(+)DTBZ) is a recently developed PET tracer to investigate the vesicular monoamine transporter type 2 (VMAT2) activity in measuring dopaminergic degeneration *in vivo* and monitoring the severity of Parkinson’s disease (PD). However, manual drawing of the striatal regions is time consuming and prone to human bias. In the current study, we developed an automated method to quantify the signals of the striatum on ^18^F-(+)DTBZ images. 39 patients with PD and 26 controls were enrolled. Traditional brain magnetic resonance imaging (MRI) and ^18^F-(+)DTBZ PET were acquired. Both indirect normalization of native PET images to the standard space through individual brain MRI and directly coregistration of native images to the transporter-specific PET template in standard space were performed. Specific uptake ratios (SURs) in 10 predefined regions were used as indicators of VMAT2 activities to correlate with motor severity. Our results showed patients with PD had significant lower SURs in the bilateral putamina, caudates and globus pallidi than controls. SURs in the caudate and putamen were significantly correlated with motor severity. The contralateral putaminal region performed best in discriminating between PD patients and controls. Finally, the results from the application of the ^18^F-(+)DTBZ PET template were comparable to those derived from the traditional MRI based method. Thus, ^18^F-(+)DTBZ PET imaging holds the potential to effectively differentiate PD patients from controls. The ^18^F-(+)DTBZ PET template-based method for automated quantification of presynaptic VMAT2 transporter density is easier to implement and may facilitate efficient, robust and user-independent image analysis.

## Introduction

Parkinson’s disease (PD) is a common neurodegenerative disorder in aging populations. According to a recent study, the annual incidence of PD was 36–49 per 100,000 person-years and prevalence in 2010 was 308–410 per 100,000 persons in the population as a whole^1^. The diagnosis of PD is mainly based on the cardinal motor features, including bradykinesia, resting tremor, cogwheel rigidity and postural instability^2,3^. The pathophysiological mechanisms of PD remain largely unknown. Nevertheless, the primary neurotransmitter deficit appears to be the loss of dopaminergic neurons in the substantia nigra (SN), and subsequently decreased axons projected to the striatum^4^. A noninvasive neuroimaging method with radiotracers targeting the dopaminergic system from positron emission tomography (PET), such as ^18^F-Dopa or ^11^C-raclopride, is helpful to evaluate the deficiency in the dopaminergic system and correlate with disease severity or clinical symptoms^5,6^. Recently, a novel tracer of [^18^F]9-fluoropropyl-(+)-dihydrotetrabenazine (^18^F-(+)DTBZ) for vesicular monoamine transporter type 2 (VMAT2) imaging with a longer half life (*t*_1/2_ = 110 minutes compared with 20 minutes of C-11) has been developed^7^. A prior study has shown that ^18^F-(+)DTBZ PET imaging is highly sensitive in detecting the VMAT2 level at the nigrostriatal terminal, an indicator of the integrity of the dopaminergic system^8–10^.

Dopaminergic deficit shown on ^18^F-(+)DTBZ PET imaging can be estimated using an uptake ratio between the region of interest (ROI, e.g., striatum) and the reference region (e.g., occipital lobe). The common approach is to engage experienced radiologists to manually draw multiple ROIs and the reference region on the corresponding structural magnetic resonance images (MRIs). However, such an approach is labor-intensive, time-consuming, and prone to human bias^11^. To overcome the limitations, atlas-based automated imaging analysis has been proposed in the perfusion like PET image in neurodegenerative studies^12,13^. This approach provides a more sophisticated analysis method to estimate regional brain activity, thereby potentially improving the reliability of data analysis. Yet, the intensity distribution in transporter-specific PET image may completely differ from that in the perfusion like PET image and/or structural MR image, which may have an impact on the performance of coregistration method^14^. This discrepancy results from the inter-modality coregistration algorithm uses a cost function based on minimizing the sum of squared differences of voxel values between the MR and the PET images. A possible solution is to make use of the transporter-specific template instead of MR image for coregistration method^15^. Recently, a specific template has been developed for various neurodegenerative disease studies^14,16,17^. Another method is to use a corresponding perfusion like PET image as an intermediate step for coregistration to the MR images and subsequently applying this transform matrix to the native PET images. This approach may offer more reasonable and accurate results given that both perfusion like PET and MR images share a similar intensity profile^18,19^.

^18^F-(+)DTBZ PET imaging could provide the perfusion like PET images and transporter-specific PET images at the same scanning session. The scanning time window for ^18^F-(+)DTBZ PET image has been well studied and the results showed that the optimal time window for measuring dopaminergic system integrity via the standardised uptake value ratio was at 90–100 minutes post-injection (i.e., the late phase image)^10^. By contrast, a perfusion-like image acquired within the first 10 minutes after injection of the tracer (i.e., the early phase image) can be used as an intermediate image for MR image coregistration^18^. Previous ^11^C raclopride studies have used the transporter-specific template to quantify dopaminergic activities^14,15^, whilst there has not been any study employing atlas-based analysis on ^18^F-(+)DTBZ PET imaging. As such, the aim of this study was to compare performance between using the early phase image as an intermediate step in coregistration with MR image and the directly normalized late phase PET image to transporter–specific PET template.

## Materials and Methods

### Subjects

A total of sixty-five participants comprising 26 healthy controls and 39 patients with PD were included in this study. The study protocol was approved by the institutional review board of the Chang Gung Memorial Hospital (CGMHIRB No. 98-2160A/98-3626A), and written informed consent was obtained from all participants prior to the study procedure. All methods were performed in accordance with the relevant guidelines and regulations. Neurologic examinations were performed on all participants. In PD patients, disease severity was assessed by the Modified Hoehn-Yahr stage and the Part III of the Unified Parkinson Disease Rating Scale (UPDRS-III) in the off-medication state, such that patients refrained from taking any antiparkinsonian medications at least 12 hours before clinical testing^20,21^. Similarly, to avoid the transient effects of dopamine-mimic drugs on vesicular dopamine levels and VMAT2 availability, imaging acquisition was performed in the off-medication state.

### Imaging acquisition

^18^F-(+)DTBZ was prepared and synthesized at the cyclotron facility of Chang Gung Memorial Hospital^22,23^. All participants were studied in a Biograph mCT PET/computed tomography system (Siemens Medical Solutions) and underwent MRI for screening of other diseases (e.g., hemorrhages and dementia) and performing spatial normalization with PET images. Brain MRI was acquired on a 3 T Siemens Magnetom TIM Trio scanner (Siemens Medical Solutions) for detailed anatomical image. A high resolution T1-weighted image was acquired with the following parameters: TR/TE: 2000/2.63 milliseconds; NEX: 1; voxel size: 1.0x1.0x 1.0 mm^3^. After injection of a mean of 386 MBq (SD = 11) of ^18^F-(+)DTBZ, an early phase (the first 10 minutes after injection) perfusion like PET image was acquired in a 3-D mode, followed by a late phase image (i.e., a single 10-minute PET scan) acquired in a 3-D mode 90 minutes after injection^10^. PET images were then reconstructed using 3-D ordered-subset expectation maximization algorithm (4 iterations, 24 subsets; Gaussian filter: 2 mm; zoom: 3) with computed tomography–based attenuation correction and with scatter and random correction provided by the manufacturer. The reconstructed images had a matrix size of 400 × 400 × 148 and a voxel size of 0.68 × 0.68 × 1.5 mm^3^.

### Image analysis

**Figure 1:**
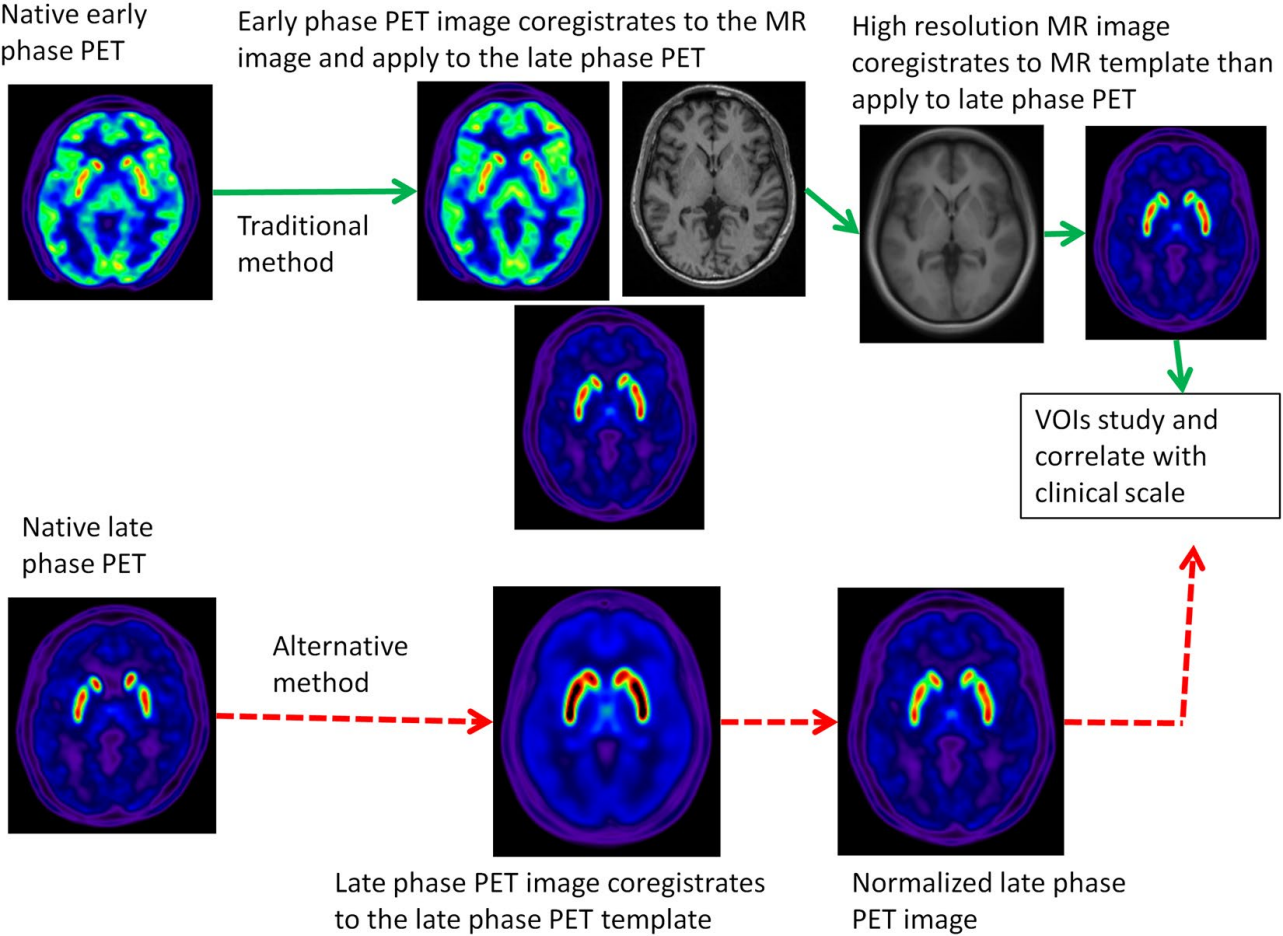
Schematic representation of the analysis process for the late phase ^18^F-DTBZ PET image.

All image data were transformed into NIFTI (*N*euroimaging*I*n*f*ormatics*T*echnology*I*nitiative) format by MRIcron tool (http://www.mccauslandcenter.sc.edu/mricro/mricron/) for further processing. In each subject, there were three images in native space for analysis, namely the early phase ^18^F-(+)DTBZ PET image, the late phase ^18^F-(+)DTBZPET image and the traditional MR image. In this work, we evaluated the correlation between clinical symptoms and the specific uptake ratios (SURs) of the selected ROIs in spatially normalized late phase ^18^F-(+)DTBZ PET images. Two normalization methods for the late phase ^18^F-(+)DTBZ PET image were compared (Fig. 1). The traditional method (i.e., MR method) used the native late phase ^18^F-(+)DTBZ PET image and performed the rigid-body coregistration to the early phase ^18^F-(+)DTBZ PET image in SPM8 (http://www.fil.ion.ucl.ac.uk/spm/software/spm8/)^24^. This process generated spatially aligned early and late phase ^18^F-(+)DTBZ PET images. The early phase ^18^F-(+)DTBZ PET image, a perfusion-like image, was then used to perform the inter-model affine coregistration with the MR image. The transform matrix was applied to the spatially aligned late phase ^18^F-(+)DTBZ PET image. This procedure produced the early and late phase ^18^F-(+)DTBZ PET images in alignment with the MR image. Finally, the high resolution MR images in native space were normalized to the Montreal Neurological Institute (MNI) standard space by SPM8 DARTEL toolbox^25^. The transform matrix was also applied to the early and late phase ^18^F-(+)DTBZ PET images. We selected control subjects and applied the aforementioned method to create the spatially normalized and transporter-related late phase ^18^F-(+)DTBZ PET template. The mean activity in the basal ganglia was normalized to a value of 1 to ensure that each subject contributed equally to the final template. The alternative method (transporter specific method) just directly used the native space late phase ^18^F-(+)DTBZ PET image and performed spatial normalization to the late phase ^18^F-(+)DTBZ PET image template. The advantages of this method were no need to include individual MRI images for spatial normalization, and the use of the transporter-specific template rather than other PET template (such as FDG-PET template) for group study. Finally, the regional SURs were calculated with the following equation: (target uptake − reference uptake)/ reference uptake. The averaged intensity in the pons and the whole occipital lobes were used as the reference regions for early phase and late phase ^18^F-(+)DTBZ PET images, respectively. Ten ROIs, including the bilateral caudate nuclei, anterior/posterior putamina, nucleus accumbens and substantia nigra, were selected for the current study.

### Statistical analysis

All statistical analyses were performed using SPSS (version 21.0). Continuous variables were expressed as the means ± standard deviations (SD). Independent t-tests and chi-square tests were performed to compare the patients’ ages and gender between controls and PDs. The opposite hemisphere of the symptomatic onset hand was defined as the contralateral side and all the patients’ images were flipped to the same symptomatic side to compare with the contra-lateral side in the same anatomical regions with independent t-tests. To determine the discriminative abilities of the mean SURs in the caudate, anterior and posterior putaminal regions between patients with PD and controls, we generated the ROC curve to investigate the accuracy and calculated areas under the curve (AUCs) for each region. Correlation analyses using Spearman’s correlation coefficient were conducted to study the relationship between the two methods in different regions. A non-linear sigmoid regression analysis was performed to find the association with the clinical UPDRS scores. Statistic significance was defined as a p-value < 0.01.

## Results

### Demographic characteristics

Patients and controls were demographically well matched as no significant group differences were found in age (mean ± SD in controls vs patients with PD: 56.0 ± 7.5 vs 55.8 ± 8.6; p = 0.93) and gender (Table 1). In the PD group, mean total UPDRS scores was 26.1 (SD = 22.5) and the median of Hoehn and Yahr stage was 1 (range 1–5).

**Table 1:** Demographical descriptions of controls and patients with Parkinson’s disease (PD).

Items	Controls (N = 26)	PD (N = 39)	P-value
Age (years)	56.0 + 7.5	55.8 + 8.6	0.93
M:F	13:13	20:19	0.92
Mean total UPDRS scores	—	26.1 + 22.5	
UPDRS-I	—	1.2 + 1.4	
UPDRS-II	—	7.3 + 6.6	
UPDRS-III	—	16.2 + 14.1	
Hoehn and Yahr stage	—	1.8 + 1.1	

### Regional differences between controls and patients with PD in early and late phase ^18^F-(+)DTBZ PET images

**Figure 2:**
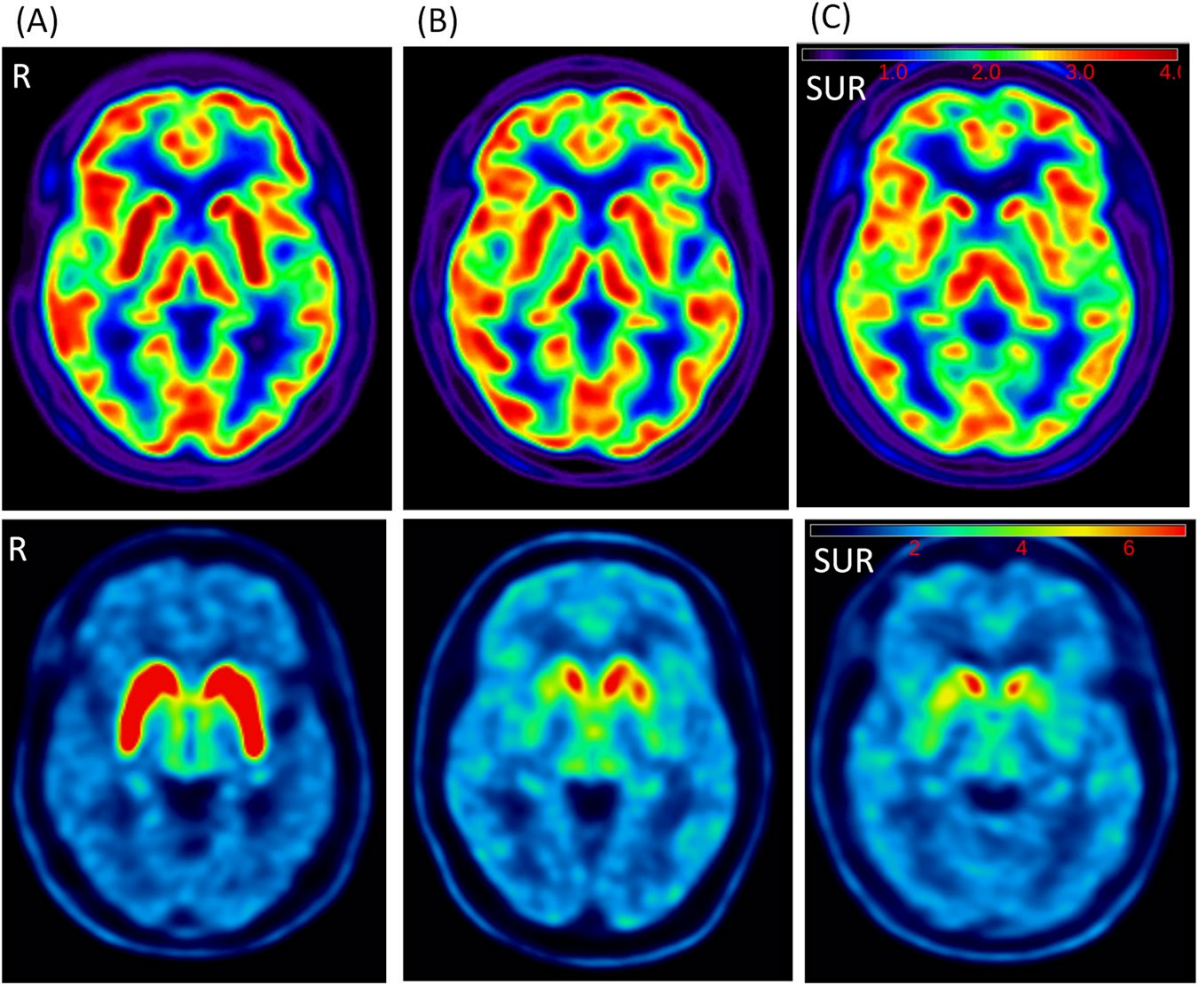
Examples of the early phase (upper row) and late phase (lower row) of ^18^F-DTBZ PET images of a control (**A**), a patient with mild PD (**B**) and a patient with severe PD (**C**). Tracer uptake was retained in the bilateral caudate and left anterior putamen regions in mild PD (Hoehn and Yahr stage = 1), but was limited to a partial region of the caudate in severe PD (Hoehn and Yahr stage = 4). Colorbar scale represents as the SUR values. R:right side.

Figure 2 presents example images of the early and late phase of ^18^F-(+)DTBZ PET images of a control, a patient with mild stage of PD (Hoehn and Yahr stage = 1) and a patient with severe stage of PD (Hoehn and Yahr stage = 4), respectively. Among the 10 selected ROIs, significantly lower mean SURs were found in the bilateral posterior putamen, ipsilateral anterior putamen and contralateral substantia nigra regions (ps < 0.001, Table 2) in the early phase images. Compared with controls, patients showed significantly lower mean SURs in the bilateral caudate, anterior and posterior putamina, and substantia nigra in the late phase images (ps < 0.001, Table 3).

**Table 2:** Regional intensity differences between controls and patients with PD in the early phase ^18^F-(+)DTBZ images.

Region	Controls	PD	P-value
Contralateral Caudate	1.35 + 0.18	1.18 + 0.26	<0.01
Ipsilateral Caudate	1.36 + 0.18	1.19 + 0.24	<0.01
Contralateral anterior putamen	1.78 + 0.18	1.57 + 0.15	<0.01
Ipsilateral anterior putamen	1.74 + 0.18	1.62 + 0.16	<0.01
Contralateral posterior putamen	1.71 + 0.20	1.17 + 0.14	<0.01
Ipsilateral posterior putamen	1.68 + 0.20	1.51 + 0.14	<0.01
Contralateral Nuclear Accumben	1.54 + 0.15	1.62 + 0.19	0.09
Ipsilateral Nuclear Accumben	1.61 + 0.16	1.61 + 0.17	0.98
Contralateral Substantia nigra	1.25 + 0.14	1.16 + 0.13	<0.01
Ipsilateral Substantia nigra	1.27 + 0.13	1.19 + 0.13	0.02

**Table 3:** Regional intensity differences between controls and patients with PD in the late phase ^18^F-(+)DTBZ images.

Region	Controls	PD	P-value
Contralateral Caudate	4.03 + 0.64	2.67 + 0.94	<0.01
Ipsilateral Caudate	4.08 + 0.66	3.11 + 1.04	<0.01
Contralateral anterior putamen	4.99 + 0.73	2.29 + 0.46	<0.01
Ipsilateral anterior putamen	4.89 + 0.74	2.77 + 0.76	<0.01
Contralateral posterior putamen	5.21 + 0.92	1.76 + 0.33	<0.01
Ipsilateral posterior putamen	5.11 + 0.89	2.06 + 0.70	<0.01
Contralateral Accumben	3.80 + 0.54	3.66 + 0.73	0.41
Ipsilateral Accumben	3.96 + 0.52	3.87 + 0.74	0.59
Contralateral Substantia nigra	2.58 + 0.29	2.03 + 0.30	<0.01
Ipsilateral Substantia nigra	2.63 + 0.33	2.17 + 0.35	<0.01

### ROC curve analysis of mean SURs from the early and late phase ^18^F-(+)DTBZ PET images in the caudate, anterior and posterior putamina between patients with PD and controls

**Figure 3:**
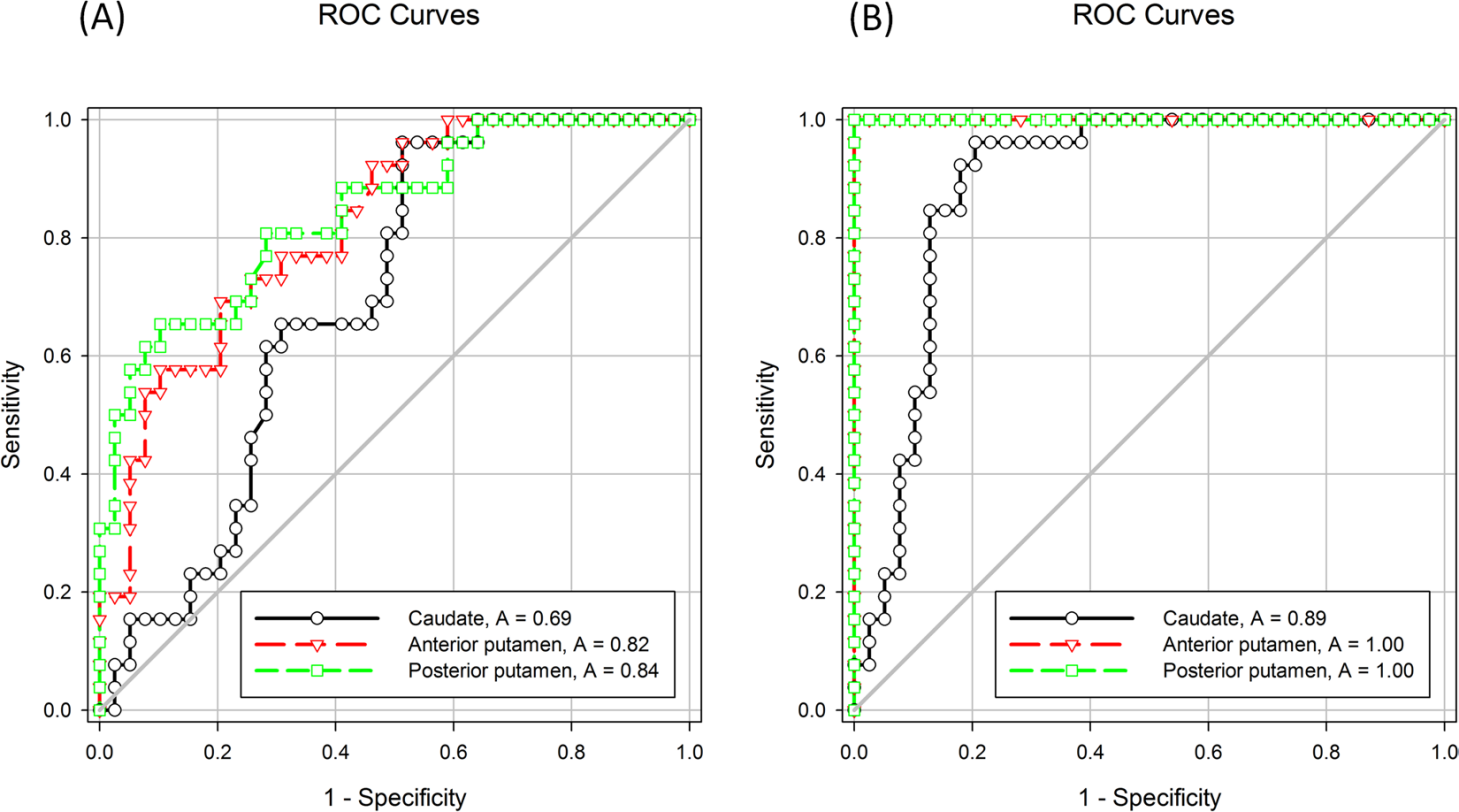
The ROC curve of the contralateal regional SURs in the early (**A**) and late (**B**) phase of ^18^F-DTBZ PET image using the traditional method.

In early phase ^18^F-(+)DTBZ PET images, ROC curves were generated using mean SURs from the contralateral caudate, anterior putamen and posterior putaminal regions for all participants by traditional method. The highest AUC value was found in the contralateral posterior putamen (AUC = 0.84), which indicated better discriminative ability than other regions (Fig. 3A). In the ipsilateral regions, the AUC of the posterior putamen (AUC = 0.75) was lower than the contralateral same region. Among the late phase ^18^F-(+)DTBZ PET images, the highest AUC value was found to be 1 in the contralateral anterior and posterior putaminal regions (Fig. 3B). These results showed that the late phase ^18^F-(+)DTBZ PET images in the anterior and posterior putaminal regions best differentiated between patients with PD and controls. For the alternative method, the AUC values were 0.82, 0.99 and 1.0 for the contralateral caudate, anterior putamen and posterior putamen regions, respectively.

### Correlation analysis in different ROIs between both methods

We compared correlation values between the traditional method (coregistrating the late phase ^18^F-(+ )DTBZ PET images to individual MRI which was subsequently transformed to MNI space) and the alternative method (directly coregistrating the late phase ^18^F-(+)DTBZ PET image to the receptor-specific template) from different ROIs. The Spearman’s correlation coefficient values in each ROI showed significant correlations between the two methods (ps < 0.01, Table 4) with varied correlation coefficient values ranging from 0.75 to 0.97. The lowest and highest values were found in the ipsilateral nuclear accumben and the ipsilateral anterior putamen, respectively. The averaged Spearman’s correlation coefficient value was 0.91. All regions showed correlation coefficient values larger than 0.9, except the bilateral nuclear accumben.

**Table 4:** Spearman’s correlation coefficient values in different ROIs between the traditional method and alternative method.

Region	Contralateral side	P-value	Ipsilateral side	P-value
Caudate	0.95	<0.01	0.91	<0.01
Anterior putamen	0.97	<0.01	0.97	<0.01
Posterior putamen	0.95	<0.01	0.93	<0.01
Nuclear accumben	0.78	<0.01	0.76	<0.01
Substantin nigra	0.94	<0.01	0.92	<0.01

### Correlations between regional SURs from the late phase ^18^F-(+)DTBZ PET image and UPDRS-III

**Figure 4:**
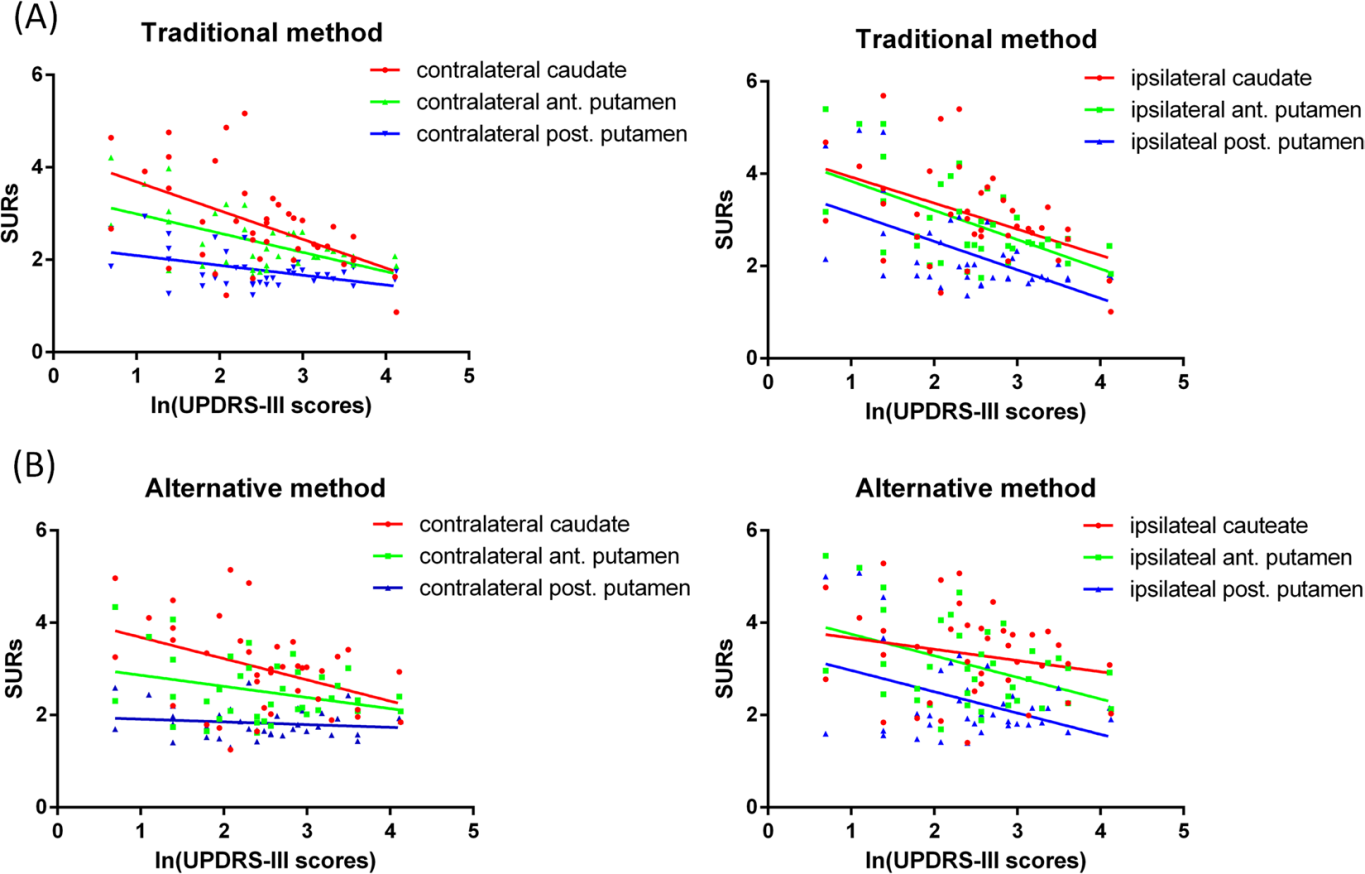
Correlations between log-transformed UPDRS-III scores and SURs in the caudate, anterior and posterior putaminal regions derived from the traditional (**A**) and alternative (**B**) methods.

**Figure 5:**
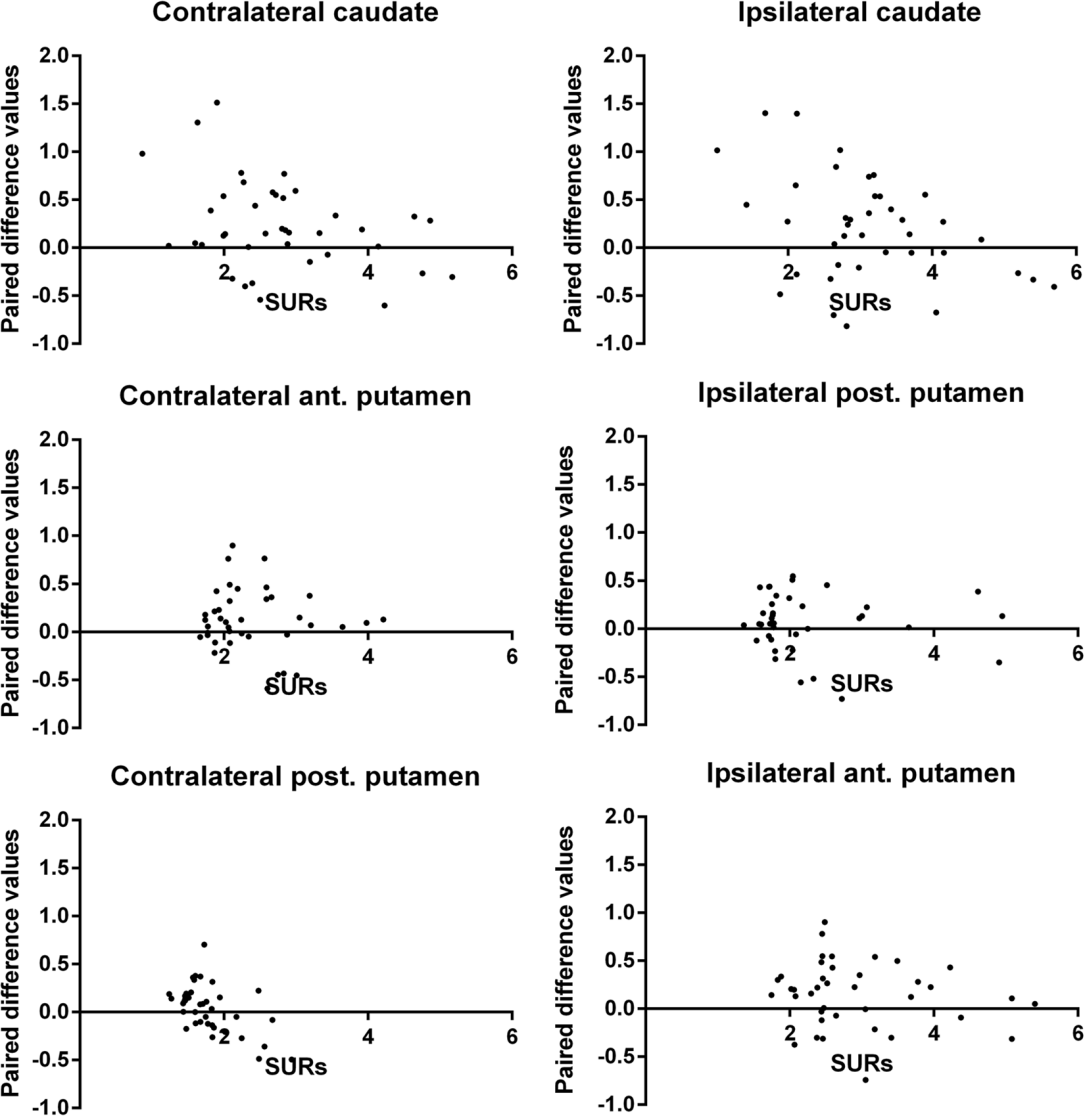
Scatter plots of the paired difference values (values derived from the alternative method subtracting from those derived from the traditional method) corresponding with the regional SURs from traditional method in various regions.

We selected SURs from the bilateral caudate, anterior and posterior putaminal regions to correlate with UPDRS-III scores (Fig. 4). Findings from the traditional method revealed that the SURs in the contralateral caudate, anterior and posterior putamina significantly correlated with log-transformed UPDRS-III scores (R square = 0.27, 0.31 and 0.21, respectively, ps < 0.01). In the ipsilateral side, the correlations remained significant (R square = 0.33, 0.34 and 0.34, respectively, ps < 0.01). Findings from the alternative method indicated that the SURs in the contralateral caudate showed a significant correlation with log-transformed UPDRS-III scores (R square = 0.17, p < 0.01), whilst in the ipsilateral side, the SURs in the anterior and posterior putamina showed significant associations with log-transformed UPDRS-III scores (R square = 0.18 and 0.18, respectively, ps < 0.01). To investigate the SURs differences between the alternative method and the traditional method, we plotted the paired differences of SURs, i.e. the alternative method subtracting from the convention method verse corresponding SURs from the traditional method in the bilateral caudate, anterior and posterior putamina (Fig. 5). Our results showed most of the positive SURs differences came from the lower SURs regions from the traditional method in the contralateral anterior and posterior putaminal regions, which suggested over-estimated SURs from the alternative method.

## Discussion

Several main findings yielded from this study. First, patients with PD exhibited significant lower mean SURs in the bilateral posterior putaminal and ipsilateral caudate regions shown on the early phase images. In the late phase ^18^F-(+)DTBZ PET images, PD patients had more extensive regions showing decreased mean SURs than controls, including the bilateral caudate, anterior putamen, posterior putamen, globus pallidum and substantia nigra. Second, the late phase images in the anterior and posterior putamina demonstrated the best discriminative ability between patients with PD and controls. Third, SUR results from the traditional and alternative methods showed significant correlations. The mean SURs in the bilateral caudate and putaminal regions also showed significant correlations with motor dysfunction measured with the UPDRS-III. These findings suggested that ^18^F-(+)DTBZ PET imaging could effectively differentiate patients with PD and controls; in addition, deficits in the dopaminergic system were found in the caudate and putaminal regions, which was in good agreement with the pathophysiological process of PD observed previously^26–29^.

### Early and late phase ^18^F-(+)DTBZ PET image characteristics in patients with PD

Prior studies have reported decreased^30^, increased^31,32^, or unchanged^33^ striatum perfusion changes in patients with PD, compared with controls. However, there is a lack of research endeavor to simultaneously measure perfusion images and dopaminergic activity images in PD. In the current study, the bilateral posterior putamina of PD patients exhibited significantly lower SURs in the early phase of ^18^F-(+)DTBZ PET image, as opposed to those of controls. Meanwhile, the spatial extension of decreased perfusion regions was more limited than that of regions showing decreased dopaminergic activity. In the late phase of ^18^F-(+)DTBZ PET images, our study showed significantly lower SURs in the caudate, putamen, globus pallidum and substantia nigra in PD patients than in controls, suggesting presynaptic nigrostriatal dysfunction in PD^34^. Using ROC curve analysis to study the discriminative ability of the striatum on the early and late phase images revealed that SURs in both anterior and posterior putamina shown on the late phase ^18^F-(+)DTBZ PET image had the highest discriminative ability between patients with PD and controls (AUC = 1).

### Generation of late phase ^18^F-(+)DTBZ PET template and comparison with the traditional method

In the current study, a late phase ^18^F-(+)DTBZ PET template generated using the corresponding high resolution MR data from healthy volunteers facilitated direct quantification of SUR ^18^F-(+)DTBZ PET binding in the putamen and caudate nucleus (striatum). Quantitative measures of presynaptic VMAT2 density in comparison to the reference values are recommended for the detection of early-stage movement disorders and the establishment of a reliable diagnosis. ^18^F-(+)DTBZ binding is typically assessed between 90 to100 min after tracer injection. At this time point, tracer binding is limited to VMAT2 regions, including the caudate nucleus and putamen, making automatic coregistration of the PET data to the corresponding MR highly inaccurate. Conversely, perfusion-like images from early timeframes of PET imaging are more suitable for image co-registration with morphological MRI^18,35^ despite that acquiring MR imaging in addition to receptor binding images from the late time frames of PET imaging is not cost-effective in most clinical settings.

In our study, we developed the late phase ^18^F-(+)DTBZ PET template allowing for direct computation of SURs in different ROIs after a single normalization step. This procedure facilitates automated imaging processing and data analysis. One-step coregistration of the late phase ^18^F-(+)DTBZ PET image to the binding template may also reduce the risks of errors introduced by the multi-step MR-based processes. Furthermore, user-independent data quantification reduces intra- and inter-operator variations that could hinder the reproducibility of the measurements. Findings from our study demonstrated that PET template-based normalization method was significantly correlated with MR-based method in each ROI (the averaged Spearman’s rho value = 0.91), indicating strong correlations between two methods^36^ and thus supporting the utilization of PET template-based normalization method as an alternative means for automated analysis, especially in the event of no available corresponding MR images.

### Regional SURs changes associated with motor dysfunction

Following the traditional method, the SURs in the caudate, anterior and posterior putaminal regions showed significant correlations with UPDRS-III scores in both contralateral and ipsilateral sides. These results demonstrated the association between the quantified striatal VMAT2 activity and motor dysfunction. Of note, with the alternative method, the SURs of the contralateral caudate, ipsilateral anterior and posterior putaminal regions showed significant correlations with UPDRS-III scores. The discrepancy may result from the relatively increased values of SURs in the regions originally showing lower SURs as the alternative method may force the lower uptake regions to fit for the intensity distribution of normal transporter-specific PET template during the normalization process, leading to over-estimated SURs in patients with more severe motor dysfunction. The application of this alternative method in severe stages of PD would require caution. Future study including adjustment of coregistration parameters or developing disease specific templates may help to resolve this issue.

### Limitation

Several limitations in this study should be mentioned. First, our patients with PD were in relatively mild stages as the median of Hoehn and Yahr stage was 1. Even in this condition, the mean SUR from the contralateral side in the posterior putamen region is only 34% compared with the ipsilateral side. Considering that our alternative method may introduce over-estimation of SURs in the lower intensity regions, we did not fine-tune our setting parameters during the normalization process. Further study should explore the different settings of the parameters during normalization process. Second, the majority of our patients were in the mild stage, except 7 cases with Hoehn and Yahr stage ≥3, which might limit the application of our alternative method to patients in moderate to severe stages. As pointed out previously that over-estimated SURs from the alternative method were found in the lower SURs regions, therefore implying that patients in moderate to severe stages might have overestimated SUR findings. Further studies focusing on testing the performances of our alternative method in moderate to severe PD cases or even creating the templates for different severities of PD are needed. Third, as we did not include detailed neuropsychological measurement in the study, our results could be confounded by other pathologies, such as Lewy body dementia. Longitudinal follow-up clinical features or neuropathological results of this cohort would be needed to verify our findings.

## Conclusion

Our findings suggested that ^18^F-(+)DTBZ PET image could early detect presynaptic VMAT2 dysfunction in patients with PD and the putaminal region could effectively discriminate between patients with PD and controls. The intensity changes in PD-associated regions were correlated with motor dysfunction. The automated imaging analysis process involving the transporter specific PET template in the current study showed highly similar results to those derived from the traditional method in estimating regional SURs, discriminating PD cases from controls and correlating with the clinical symptoms. Further studies are needed to test the validity of our alternative method in a larger cohort comprising patients with moderate to severe stages of PD.

## Data Availability

Additional clinical data are available from laboratory studies. Please contact Dr. Kun-Ju Lin (E-mail: kunjulin@gmail.com) if this information is of interest.
